# Optimizing Diet to Slow CKD Progression

**DOI:** 10.3389/fmed.2021.654250

**Published:** 2021-06-25

**Authors:** Pablo Molina, Eva Gavela, Belén Vizcaíno, Emma Huarte, Juan Jesús Carrero

**Affiliations:** ^1^Department of Nephrology, Hospital Universitari Dr. Peset, Fundación para el Fomento de la Investigación Sanitaria y Biomédica de la Comunitat Valenciana (FISABIO), Valencia, Spain; ^2^Department of Medicine, Universitat de València, Valencia, Spain; ^3^Department of Nephrology, Hospital San Pedro, Logroño, Spain; ^4^Department of Medical Epidemiology and Biostatistics, Karolinska Institutet, Stockholm, Sweden

**Keywords:** chronic kidney disease, protein restricted diet, nutrition, salt restriction, renoprotection

## Abstract

Due to the unique role of the kidney in the metabolism of nutrients, patients with chronic kidney disease (CKD) lose the ability to excrete solutes and maintain homeostasis. Nutrient intake modifications and monitoring of nutritional status in this population becomes critical, since it can affect important health outcomes, including progression to kidney failure, quality of life, morbidity, and mortality. Although there are multiple hemodynamic and metabolic factors involved in the progression and prognosis of CKD, nutritional interventions are a central component of the care of patients with non-dialysis CKD (ND-CKD) and of the prevention of overweight and possible protein energy-wasting. Here, we review the reno-protective effects of diet in adults with ND-CKD stages 3–5, including transplant patients.

## Introduction

Advanced chronic kidney disease (CKD) is a systemic disorder which is associated with high mortality and poor quality of life. Different treatments and lifestyle modifications are needed to avoid progression to kidney failure, which requires of kidney replacement therapy (maintenance dialysis or transplantation), and exceedingly costly therapy to Society (1–3). Chronic kidney disease progression is largely conditioned by hemodynamic and metabolic factors independent of the primary kidney disease, many of them, such as the high blood pressure (BP), the hyperfiltration, or the proteinuria are highly influenced by diet (4). Moreover, due to the kidney's unique role in nutrient metabolism, patients with advanced CKD are unable to maintain adequate nutrient homeostasis, developing metabolic disorders as sodium and volume overload, hyperkalemia, hyperphosphatemia, metabolic acidosis, altered hormone regulation, and inflammation. Accordingly, nutritional interventions should be a fundamental strategy in the treatment of patients with CKD (5–7).

In recent years, several studies, trials, and meta-analyses have evidenced the effectiveness of protein restriction and others nutritional interventions on kidney outcomes (8–17). This evidence was judged to increase the strength of recommendations for the nutritional management of patients with CKD in the 2020 update of the KDOQI guidelines (18). In this review, we aim to summarize recent studies on the role of diet, focusing on salt and protein restriction, as well as the use of supplements with essential amino acids (AAs) and keto analogs (KAs) to delay the progression of CKD and, at the same time, to preserve the nutritional status of patients with ND-CKD.

## Dietary Requirements in Patients With Kidney Disease

Table 1 shows current recommendations for nutritional requirements in adult patients with non-dialysis CKD (ND-CKD), and kidney transplant recipients (KTR) (18, 19). A caloric intake of 25–35 kcal/kg/day is recommended to counteract the excess resting energy expenditure secondary to inflammation and comorbidities, as well as for preserving a neutral or positive nitrogen balance. However, this recommendation should be individualized according to the patient's profile, including age, lean body mass (which is the primary determinant of energy expenditure), physical activity, and the underlying etiology of kidney disease (20, 21). According to the 2020 KDOQI guidelines, the recommended protein intake for stable patients with ND-CKD 3–5 dialysis is 0.55–0.60 g/kg/day, which can be reduced to 0.28–0.43 g/kg/day if it is supplemented with 7–15 g/day of KAs and essential AAs. In the case of diabetic patients, guidelines suggest a higher protein intake up to 0.6–0.8 g/kg/day to glycemic control. Any intercurrent catabolic episode may require increasing energy and protein intake independently of CKD stage (22). Regarding protein quality, there is no consensus on whether the protein source impacts differently on the risk of CKD progression (18).

**Table 1:** Nutritional requirements for patients with non-dialysis CKD according to 2020 KDOQI Guidelines (18).

	**ND-CKD stage 3–5**	**Transplantation**
Energy (kcal/kg ideal weight/day)^a^	25–35	25–35 in maintenance KTR 25 (obesity) 35–40 for the first 4 weeks after transplantation
Protein (g/kg/day)^a^^,^^b^	0.55–0.60 or 0.28–0.43 plus keto/amino acid supplementation	0.8
	0.80–0.90 (diabetes)	0.6–0.8 (CKD stages 3–5 T)
	1.0 (illness)	≥1.4 (for the first 4 weeks after transplantation or if high doses of prednisone is required)
Sodium (g/day)	<2.3	<2.3
Potassium^c^	Adjust dietary potassium intake to maintain serum potassium within the normal range	Adjust dietary potassium intake to maintain serum potassium within the normal range
Calcium (mg/day)	800–1,000^d^	Insufficient data to define optimal dietary calcium intake in KTR (research priority)
Phosphorus^e^	Adjust dietary phosphorus intake to maintain serum phosphate levels in the normal range	Adjust dietary phosphorus intake to maintain serum phosphate levels in the normal range
Fiber (g/day)	25–38	25–38
Vitamin D (IU/day)	600–800	600–800
Vitamin B12 (μg/day)^f^	2.4	2.4
Folic acid (μg/day)^f^	400	400
Vitamin C (mg/day)^f^	90 (M), 75 (W)	90 (M), 75 (W)
Vitamin E (mg/day)^f^	15	15
Vitamin K (μg/day)^f^	120 (M), 90 (W)	120 (M), 90 (W)
Selenium (μg/day)^f^	55	55
Zinc (mg/day)^f^	11 (M), 8 (W)	11 (M), 8 (W)

*ND-CKD, non-dialysis chronic kidney disease; KTR, kidney transplant recipients; M, men; W, women*.

a *Energy and protein intake should be adapted to age, gender, level of physical activity, body composition, weight status goals, CKD stage, and concurrent illness or presence of inflammation to maintain normal nutritional status. If present, priority should be given to the correction of protein-energy wasting*.

b *Not enough evidence to make a statement on protein sources*.

c *Guidelines do not suggest specific dietary K range (restriction per se may favor other nutrient deficiencies). Before restricting healthy foods, other causes of hyperkalemia (acidosis, constipation…) should be corrected*.

d *Including dietary calcium, calcium supplementation, and calcium-based phosphate/potassium binders*.

e *When making decisions about phosphorus restriction treatment, consider the bioavailability of phosphorus sources (e.g., animal, vegetable, additives)*.

f *No specific recommendations are provided by KDOQI guidelines. In the absence of evidence specific for persons with CKD, recommended Dietary Allowances for Adult General Population should apply*.

Kidney transplant recipients requires a different nutritional management depending on the post transplantation period. During the perioperative period, KTR need to adequate their intake of energy to 35–40 kcal/kg/day and of proteins up to 1.4 g/kg/day for at least 4 weeks (19) to compensate the increase in protein catabolism subsequent to the use of steroid and surgical stress. However, in the maintenance phase, the goal is to optimize the nutritional status with a slight reduction of the caloric intake down to 30 kcal/kg/day. Obese KTR should reduce their caloric consumption to levels lower than their energy expenditure, being values close to 25 kcal/kg/day an adequate approximation (19). Due to the lack of available studies in this population (23), it has been proposed that those with normal kidney function should follow similar recommendations to the general population, whereas for KTR with chronic allograft dysfunction, it is recommended to provide a protein-restricted diet just as in ND-CKD (19).

A modest sodium restriction (<2.3 g/day) is recommended for the management of CKD patients to achieve better volume control, reducing BP, and proteinuria synergistically with available pharmacologic interventions (18). A daily fiber intake of 25–30 g/day or more for CKD patients may be suggested, being this amount similar to recommendations for the general population (7). Potassium restriction in CKD may prevent from complying with this recommendation but in general terms CKD patients do not require aggressive dietary potassium restriction until advanced stages or if hyperkalemia risk is judged high (24–26). Recently, it has been suggested to avoid high potassium foods with poor nutritional value (i.e., bran products, or salt substitutes) and correct other causes of hyperkalemia, such as metabolic acidosis or use of renin-angiotensin-aldosterone system (RAAS) inhibitors, before restricting healthy foods (27). Acidosis is a key risk factor in the progression of CKD, being fruits and vegetables an alternative to oral alkali that may reduce the risk for volume retention and/or hypertension related to bicarbonate supplementation (28). Given the role of calcium balance and the serum phosphate in the development of cardiovascular calcifications, several experts recommend limiting total dietary calcium intake to 800–1,000 mg/day or less (including dietary calcium, calcium supplementation, and calcium-based phosphate binders) in adults with CKD 3–4 not taking active vitamin D analogs. Although phosphate intake to 800–1,000 mg/day (800–1,300 in KTR) was recommended previously (29, 30), new guidelines suggest adjusting dietary phosphorus intake to maintain serum phosphate levels in the normal range (18). Limiting processed foods with phosphorous-based additives and encouraging home-cooked meals from fresh ingredients (preferably plant-based foods) should be the first-line interventions for phosphorus restriction (31). As in the general population, vitamin D intake for CKD patients is recommended at 600–800 IU/day, but the optimal vitamin D levels in serum remain controversial (31, 32).

## Practice Strategies: Need for Individualizated and Not too Restrictive Diet

Because a “kidney” diet comes with many restrictions, adherence to such a diet can be difficult and problematic (5). Too many restrictions should be avoided (18), as they can lead to poor intake. Modifications in diet are rarely required for patients with a GFR ≥60 ml/min/1.73 m^2^. Such patients should be advised to follow the same dietary recommendations as for the general population [low sodium and refined sugar, avoidance of red and processed meats, and high content of fruits, vegetables, legumes, fish, poultry, and whole grains (33)]. However, in the later stages of CKD, diet must be modified across the spectrum of the disease, according to the type of renal replacement therapy if any, and the presence of other comorbidities (5).

## Adequate Protein Intake to Slow CKD Progression

### Biosynthesis and Degradation of Proteins

Protein digestion includes the process of breaking down proteins into their constituent AAs, which can be used either to create proteins or as an energy source, the later especially in times of starvation (34). Unlike carbohydrates and fats, if proteins are consumed in excess, the body has no capacity for their storage. As a result, excess AAs consumed are processed, being the hydrocarbon skeletons stored as fat, while the surplus of nitrogen must be removed, in as much as nitrogenous waste products are harmful, and there are no nitrogenous compounds in energy-transduction pathways (35). This is especially relevant for CKD patients, in whom consuming diets rich in protein leads to the accumulation of nitrogenous waste products and ions, causing the uremic syndrome. Transamination reactions are reversible and can thus be used to synthesize AAs from α-ketoacids (36, 37). However, human beings cannot synthesize all of the AAs (the so-called essential AAs), which must be supplied in the diet. A deficiency of even a single AA leads to a negative nitrogen balance. In this state, more protein is degraded than is synthesized, so more nitrogen is excreted than is ingested (38). The latter is particularly relevant for CKD patients under low protein diet (LPD), who may be at risk for developing protein-energy wasting (PEW) if not adequately monitored.

### Effects of Amino Acids and Proteins on Renal Hemodynamics

**Figure 1:**
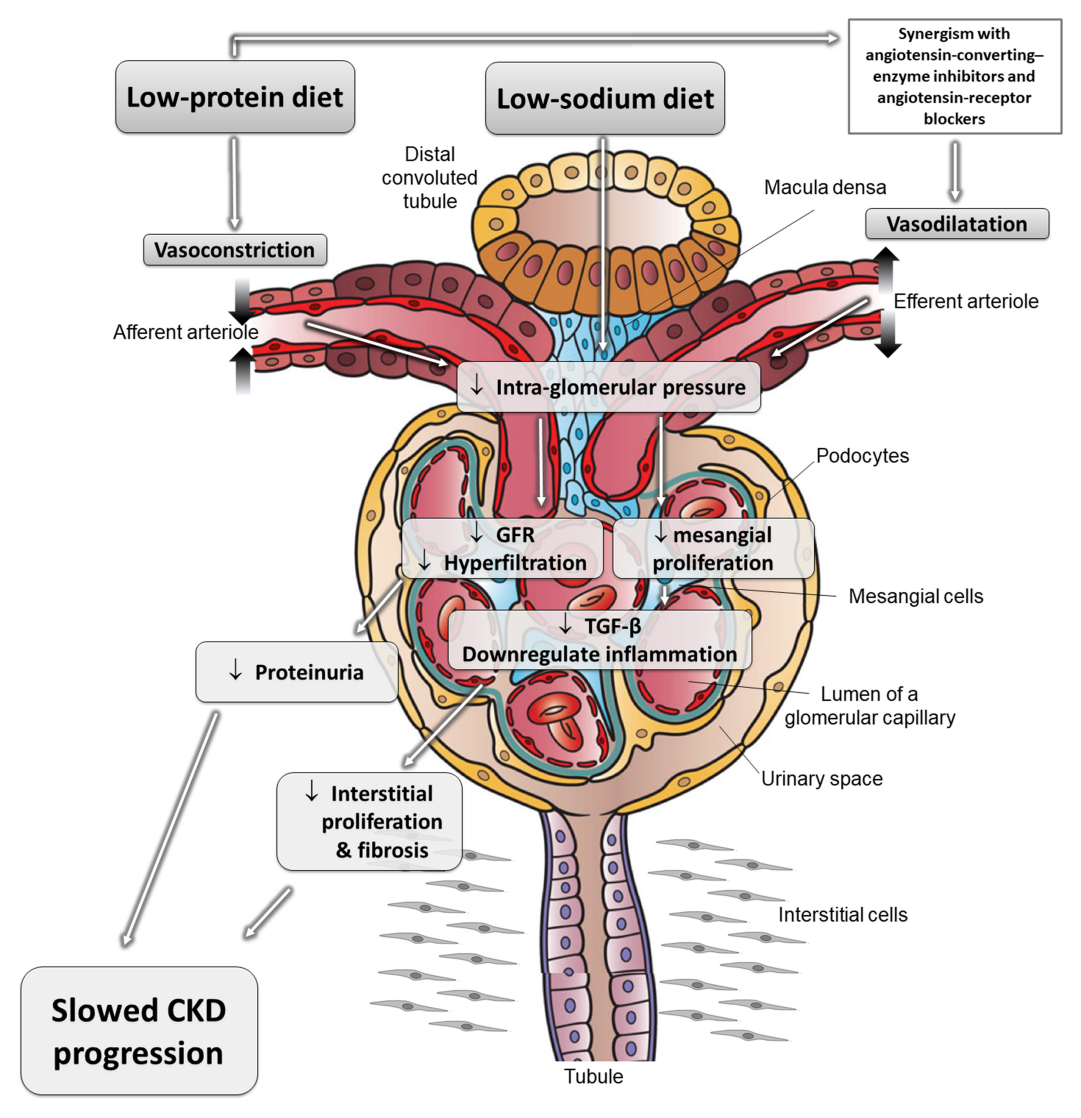
The effects of different nutritional interventions to slow progression of CKD. Schematic representation of reno-protective mechanisms related to protein and diet restriction. These effects can be synergistic with the mechanisms of angiotensin-converting-enzyme inhibitors and angiotensin-receptor blockers, which dilate the efferent arteriole and reduce intraglomerular pressure and glomerular damage. Adapted from Kalantar-Zadeh and Fouque (5). CKD, chronic kidney disease; GFR, glomerular filtration rate; TGF-β, transforming growth factor beta.

Excess nutritional load of AAs dilates the “afferent” arteriole, increasing intraglomerular pressure and resulting in “glomerular hyperfiltration” and increased renal plasma flow (39). Glomerular hyperfiltration may contribute to progression of CKD (40–43). Conversely, a lower protein intake leads to greater constriction of the afferent arteriole, resulting in a reduction in GFR (Figure 1). In addition to hemodynamic-mediated mechanisms, protein restriction may protect against CKD progression by changes in cytokine expression and matrix synthesis (5).

### Metabolic Adaptation to a Reduction in Protein Intake

Most authors agree that, in the absence of intercurrent disease, the protein requirements for patients with CKD are not substantially different from those of healthy subjects (44). In normal healthy adults, the minimum dietary protein intake to prevent negative nitrogen balance is approximately 0.6 g/kg/day. Maintaining this LPD or a very low protein diet (VLPD) of nearly 0.3 g/kg/day supplemented with AAEs and KAs are sufficient to achieve nitrogen balance and normal nutritional parameters (45, 46). As in healthy subjects, CKD patients, can improve AA utilization and nitrogen balance during LPD and VLPD by activating appropriate adaptive responses (47). These include normal anabolic responses to dietary protein restriction (suppression of AA oxidation) and feeding (stimulation of protein synthesis and inhibition of protein degradation), and the recycling of AAs derived from protein breakdown (35, 36). It is important to note that these diets require sufficient caloric intake to effectively use dietary protein (48).

### Benefits of Protein Restriction on Kidney Outcomes

The benefits of LPDs include slowing the progression of CKD and reducing uremic symptoms and metabolic disorders (20, 21, 48, 49). Nearly 20 RCTs have assessed the effects of protein restriction on several renal outcomes, including CKD progression, proteinuria, phosphate levels, acidemia, and BP, which have been summarized in several meta-analyses and two Cochrane reviews (8–11, 15, 16, 50–56). Overall, the balance of evidence suggests a benefit of dietary protein restriction. The 2020 KDOQI guidelines recommend, in non-diabetic adults with CKD 3–5 who are metabolically stable, protein restriction for reducing the risk of progression to end-stage renal disease (ESRD) and death (evidence 1A), and improve quality of life (1C). In adults with CKD 3–5 and who have diabetes, the guidelines suggest (with an evidence grade of “opinion”) a higher protein intake up to 0.8–0.9 g/kg/day (18). For the study of the effect of LPD on survival, they identified five RCTs. Three studies clearly indicated a beneficial effect of moderate restriction in dietary protein in the development of ESRD/renal death (57–60), whereas two studies did not (61, 62). The results of the secondary analysis on the number of ESRD/renal death events combined from the three positive studies indicated a beneficial effect of protein restriction (OR 0.621; 95% CI: 0.391–0.985). For the effect of LPD on quality of life, they identified a single RCT that demonstrated how the group with protein restriction presented significantly higher scores for general health and state physical compared to the control group (62). For the study of the effect of VLPD supplemented with AAEs and KAs, they reviewed a total of 13 RCTs and one non-RCT (63–76). The pooled analysis indicated a probable overall benefit of VLCD+KAs supplementation for the development of ESRD/renal death in patients with CKD stages 3–5 (RR 0.65; 95% CI: 0.49–0.85). After the 2020 K-DOQI guidelines, a new systematic review and meta-analysis explored the effectiveness and safety of VLCD supplemented with KAs compared to LPD or a normal protein diet in patients with CKD (15). Seventeen RCTs with a total of 1,459 patients were included. KAs-supplemented VLCD significantly conserved GFR and reduced proteinuria, phosphorus and parathyroid hormone levels, systolic and diastolic BP, as well as serum cholesterol. Additionally, the analysis by subgroups showed how the VLPD supplemented with KAs was superior to the LPD with KA in the rate of decrease in GFR.

Evidence for the benefits of protein restriction in KTR remains elusive (18, 19). Reasons for this limited evidence include the common requirement for a LPD of being metabolically stable, a difficult condition to achieve for patients under immunosuppressive treatment. However, many of KTR have a reduced nephron mass and, therefore, could benefit from protein restriction (77). In a crossover RCT, Rosenberg et al. examined the effects of dietary protein restriction in 14 patients with chronic kidney rejection. Low protein diet was associated with a significant improvement in plasma renin activity without any change in BP, GFR, or renal plasma flow. Studies are needed to establish the efficacy and the safe level of dietary protein restriction in KTR (78).

### Challenges and Risks for Protein Restriction Diets

Major concerns for LPD/VLPD are adherence and safety (79–81). Patients' adherence to these dietary regimens is low, being the knowledge and the satisfaction that the patients obtain from diet compliance the main determinants of adherence (82). Even if the patients are well-informed about the benefits of LPD/VLPD, some of them may find it difficult to adapt their lifestyles to the diet. Accordingly, it is of paramount importance to educate patients about the role of diet therapy with protein restriction for the treatment of CKD, taking into account their eating habits and preferences (81, 83). Regarding nutritional security, it has been definitively demonstrated that PEW is extremely rare in patients with CKD provided that the energy intake is in the normal-high range (30–35 kcal/kg/day), the protein intake is increased in case of acute illness or hospitalization, and provided that a nutritional assessment is periodically conducted (7, 18, 80). Consequently, trained personnel (ideally a registered dietitian nutritionist) is strongly recommended to develop individualized dietary programs and routinely monitor and advise patients (18, 80, 82, 84). However, this approach is time and money consuming (80). An option for those centers in which dedicated personnel (i.e., dietitian) is not available or the expertise of the nephrologist in managing diets is not optimal may be found in simplified and practical approaches to LPD (80, 85, 86). Indeed, even small reductions in protein intake as low as 0.2 g/kg/day may also delay the need for dialysis treatment (10, 56, 80). In these Nephrology Units devoid of dieticians, protein intake may be assessed by urinary urea-N excretion, whereas weight and other anthropometric methods (e.g., skinfold thickness) may be useful for indirectly monitoring caloric intake and body fat (85, 87).

### Effects of the Nature of Protein. Is a Vegetarian Diet an Option?

There is insufficient evidence to recommend a particular protein type (plant vs. animal) in terms of the effects on CKD progression or nutritional status (18). However, several observational studies have suggested that plant proteins may have more reno-protective effects than animal proteins. A diet rich in protein from plant sources may slow the progression of CKD (88–92), decrease proteinuria (93, 94), lower the level of uremic toxins (94–99), phosphorus intake, and the endogenous production of acid (89, 90, 100, 101). Moreover, that such diet could potentially improve survival (102). However, the confounding factors inherent in a diet rich in plant-based protein (i.e., higher intakes of vitamins and antioxidants) make it difficult to draw definite conclusions (103, 104). In a RCT, Garneata et al. compared a KA-supplemented vegetarian VLPD with conventional LPD in 207 CKD patients (89). The probability to reach the end point (i.e., KRT or a >50% eGFR reduction) was lower in the supplemented VLPD group than in the LPD group.

## Dietary Salt Restriction

Dietary sodium intake is a modifiable factor that can impact on the risk of CKD progression as well as on cardiovascular disease in CKD patients. Previous reports have demonstrated the effect of sodium intake on fluid overload and hypertension, both predictors of kidney progression and cardiovascular remodeling (105–109). In addition, high sodium intake might have direct toxic effects on blood vessels (109, 110). High salt intake is also a well-established risk factor for hypertension in KTR and can result in decreased graft survival (2, 111).

Conversely, salt restriction RCTs demonstrate a reduction in BP and proteinuria, with potential benefits on CKD progression and survival (5). A Cochrane review summarized the effects of salt restriction in CKD (8). Unfortunately, these studies did not show collectively a beneficial effect of a lower sodium intake on mortality, cardiovascular events, or CKD progression, probably due to their short follow-up and the limited sample size. It is interesting to highlight a significant decrease in proteinuria associated to a low salt diet, that was observed in all the RCTs that reported this outcome (112–115). The 2020 KDOQI guidelines recommend in adults with CKD 3–5 (1B), CKD 5D (1C), or postransplantation (1C), a limitation in the sodium intake to <2.3 g/d (<100 mmol/d) to achieve a BP reduction, an improvement in volume control and a decrease in proteinuria levels (2A) (18). Nevertheless, the lack of long-term RCTs assessing the effectiveness and safety of dietary salt restriction on CKD progression and survival prevents any firm conclusions about these hard outcomes.

## Reduced Phosphorus Intake

Phosphate-specific diet therapy provided by a dietitian may reduce phosphate levels in CKD, although overall certainty of evidence is low (116). However, association between hyperphosphatemia and adverse cardiovascular outcomes and CKD progression is robust in this population (117–124), also in KTR (125–127). Altogether, it seems reasonable to recommend adjusting dietary phosphorus intake to maintain serum phosphate levels in the normal range (18, 105).

## Dietary Caloric Restriction

Obesity constitutes a risk factor for diabetic and non-diabetic kidney disease (128), and there is some evidence suggesting that weight reduction through diet and lifestyle modifications could be considered as a component of the reno-protective regimen of obese patients with ND-CKD (129). Bariatric surgery reduces risk factors implicated in the progression of kidney injury in obesity and type 2 diabetes mellitus (130, 131). Dietary calorie restriction and exercise may reduce oxidative stress and inflammatory in patients with moderate to severe CKD (132), whereas weight loss may lead to better BP control and reduction of the obesity-related glomerular hyperfiltration and proteinuria (133–136).

## Fiber Intake and Probiotics

Evidence is emerging for the effects of fiber intake on uremic toxins generation (104, 137, 138). In a placebo-controlled RCT involving 30 patients with ND-CKD, total plasma p-cresol concentration was reduced by 40% after taking a synbiotic for 4 weeks (139). According to a recent meta-analysis involving eight studies of 261 patients with CKD stages 3–5D, probiotics supplementation may reduce the levels of p-cresol sulfate and elevate the levels of IL-6, thereby protecting the intestinal epithelial barrier of patients with CKD (14). However, it remains uncertain if increasing fiber intake to normalize intestinal microflora could delay CKD progression.

## Dietary Patterns and CKD Progression

Historically, research recommendations and guidelines have focused primarily on modifying the single intake of micro or macronutrients (57). However, eating habits generally remain little over time for each individual, so that the overall dietary pattern may be more decisive for patients than an excess or deficiency in one specific nutrient (103). Adherence to healthy diet patterns as the Mediterranean and the DASH (The Dietary Approach to Stop Hypertension) diets has been linked to less rapid kidney function decline and favorable effects on cardiovascular morbidity and mortality in ND-CKD patients, including KTR (140, 141). Plant-based diets could also mitigate metabolic acidosis in patients with CKD and potentially slow the progression of kidney disease, but evidence is limited (104). Conversely, a Western diet (rich in saturated fat, red and processed meat, and sweets) has been associated with an increased risk of CKD progression and albuminuria (142). The evidence is not conclusive as not all studies associate healthy dietary patterns and risk of ESRD (143). Evidence from interventional studies is also very limited (144, 145). Altogether, there is a possibility that healthy dietary patterns may prevent the development of ESRD (5, 81, 146).

## Conclusions

Close monitoring to adherence to dietary recommendations and frequent evaluation of nutritional status is fundamental in the management of patients with CKD, since it can affect important health outcomes, including CKD progression, quality of life, morbidity, and mortality. Within these nutritional measures, salt restriction, LPD and VLPD supplemented with AAs and KAs of nitrogen-free AAs, have been shown in recent meta-analyzes of RCTs to be effective in modifying the natural history of CKD, delaying the fall of the GFR, decreasing proteinuria, BP levels, or bone mineral disorder parameters, without increasing the risk of PEW. Patients' preferences and compliance have to be considered when prescribing LPD/VLPD in order to increase the adherence. Additional nutritional measures to delay CKD progression, some of them considered as experimental, may include the limitation of phosphate and calorie intake, the increase of fiber intake, and the promotion of healthy dietary patterns.

## Author Contributions

All authors listed have made a substantial, direct and intellectual contribution to the work, and approved it for publication.

## Conflict of Interest

PM acknowledges consultation or speaker fees from Abbott Nutrition, Amgen, Baxter, Fresenius-Kabi, Nutricia, Palex, Sanofi-Genzyme, and ViforPharma. JC acknowledges speaker fees from Abbott Nutrition, Baxter, Fresenius, and Nutricia. The remaining authors declare that the research was conducted in the absence of any commercial or financial relationships that could be construed as a potential conflict of interest.

## References

[1] Levey AS Atkins R Coresh J Cohen EP Collins AJ Eckardt KU. Chronic kidney disease as a global public health problem: approaches and initiatives - a position statement from Kidney Disease Improving Global Outcomes. Kidney Int. (2007) 72:247–59. 10.1038/sj.ki.5002343 17568785

[2] KDIGO clinical practice guideline for the evaluation and management of chronic kidney disease. Kidney Int Suppl. (2013) 3:1–150. 10.1038/kisup.2012.73 23989362

[3] Rebollo-Rubio A Morales-Asencio JM Pons-Raventos ME Mansilla-Francisco JJ. Review of studies on health related quality of life in patients with advanced chronic kidney disease in Spain. Nefrologia. (2015) 35:92–109 (In English, Spanish). 10.3265/Nefrologia.pre2014.Jul.12133 25611838

[4] McClellan WM Flanders WD. Risk factors for progressive chronic kidney disease. J Am Soc Nephrol. (2003) 14(7 Suppl 2):S65–70. 10.1097/01.asn.0000070147.10399.9e 12819305

[5] Kalantar-Zadeh K Fouque D. Nutritional management of chronic kidney disease. N Engl J Med. (2017) 377:1765–76. 10.1056/NEJMra1700312 29091561

[6] Carrero JJ Stenvinkel P Cuppari L Ikizler TA Kalantar-Zadeh K Kaysen G. Etiology of the protein-energy wasting syndrome in chronic kidney disease: a consensus statement from the International Society of Renal Nutrition and Metabolism (ISRNM). J Ren Nutr. (2013) 23:77–90. 10.1053/j.jrn.2013.01.001 23428357

[7] Bellizzi V Carrera JJ Chauveau P. Retarding Chronic Kidney Disease (CKD) progression: a practical nutritional approach for non-dialysis CKD. Nephrol. Point Care. (2016) 2:pocj.5000207. 10.5301/pocj.5000207

[8] Palmer SC Maggo JK Campbell KL Craig JC Johnson DW Sutanto B. Dietary interventions for adults with chronic kidney disease. Cochrane Database Syst Rev. (2017) 4:CD011998. 10.1002/14651858.CD011998.pub2 28434208 PMC6478277

[9] Rhee CM Ahmadi SF Kovesdy CP Kalantar-Zadeh K. Low-protein diet for conservative management of chronic kidney disease: a systematic review and meta-analysis of controlled trials. J Cachexia Sarcopenia Muscle. (2018) 9:235–45. 10.1002/jcsm.12264 29094800 PMC5879959

[10] Hahn D Hodson EM Fouque D. Low protein diets for non-diabetic adults with chronic kidney disease. Cochrane Database Syst Rev. (2018) 10:CD001892. 10.1002/14651858.CD001892.pub4 (Update in: *Cochrane Database Syst Rev*. (2020). 10:CD001892). 33118160 PMC8095031

[11] Yan B Su X Xu B Qiao X Wang L. Effect of diet protein restriction on progression of chronic kidney disease: a systematic review and meta-analysis. PLoS ONE. (2018) 13:e0206134. 10.1371/journal.pone.0206134 30403710 PMC6221301

[12] McMahon EJ Campbell KL Bauer JD Mudge DW. Altered dietary salt intake for people with chronic kidney disease. Cochrane Database Syst Rev. (2015) 18:CD010070. 10.1002/14651858.CD010070.pub2 25691262

[13] Garofalo C Borrelli S Provenzano M De Stefano T Vita C Chiodini P. Dietary salt restriction in chronic kidney disease: a meta-analysis of randomized clinical trials. Nutrients. (2018) 10:732. 10.3390/nu10060732 29882800 PMC6024651

[14] Jia L Jia Q Yang J Jia R Zhang H. Efficacy of probiotics supplementation on chronic kidney disease: a systematic review and meta-analysis. Kidney Blood Press Res. (2018) 43:1623–35. 10.1159/000494677 30380555

[15] Chewcharat A Takkavatakarn K Wongrattanagorn S Panrong K Kittiskulnam P Eiam-Ong S. The effects of restricted protein diet supplemented with ketoanalogue on renal function, blood pressure, nutritional status, and chronic kidney disease-mineral and bone disorder in chronic kidney disease patients: a systematic review and meta-analysis. J Ren Nutr. (2020) 30:189–99. 10.1053/j.jrn.2019.07.005 31607548

[16] Rughooputh MS Zeng R Yao Y. Protein diet restriction slows chronic kidney disease progression in non-diabetic and in type 1 diabetic patients, but not in type 2 diabetic patients: a meta-analysis of randomized controlled trials using glomerular filtration rate as a surrogate. PLoS ONE. (2015) 10:e0145505. 10.1371/journal.pone.0145505 26710078 PMC4692386

[17] Ash S Campbell KL Bogard J Millichamp A. Nutrition prescription to achieve positive outcomes in chronic kidney disease: a systematic review. Nutrients. (2014) 6:416–51. 10.3390/nu6010416 24451311 PMC3916870

[18] Ikizler TA Burrowes JD Byham-Gray LD Campbell KL Carrero JJ Chan W. KDOQI clinical practice guideline for nutrition in CKD: (2020). Update. Am J Kidney Dis. (2020) 76(3 Suppl 1):S1–S107. 10.1053/j.ajkd.2020.05.006 (Erratum in: *Am J Kidney Dis*. (2020) 77:308. 10.1053/j.ajkd.2020.05.006 32829751

[19] Chadban S Chan M Fry K Patwardhan A Ryan C Trevillian P. The CARI guidelines. Protein requirement in adult kidney transplant recipients. Nephrology (Carlton). (2010) 15(Suppl 1):S68–71. 10.1111/j.1440-1797.2010.01238.x 20591048

[20] Kovesdy CP Kopple JD Kalantar-Zadeh K. Management of protein-energy wasting in non-dialysis-dependent chronic kidney disease: reconciling low protein intake with nutritional therapy. Am J Clin Nutr. (2013) 97:1163–77. 10.3945/ajcn.112.036418 23636234 PMC3652918

[21] Chan M Kelly J Tapsell L. Dietary modeling of foods for advanced CKD based on general healthy eating guidelines: what should be on the plate? Am J Kidney Dis. (2017) 69:436–50. 10.1053/j.ajkd.2016.09.025 28129911

[22] Fiaccadori E Sabatino A Barazzoni R Carrero JJ Cupisti A De Waele E. ESPEN guideline on clinical nutrition in hospitalized patients with acute or chronic kidney disease. Clin Nutr. (2021) 40:1644–68. 10.1016/j.clnu.2021.01.028 33640205

[23] Nolte Fong JV Moore LW. Nutrition trends in kidney transplant recipients: the importance of dietary monitoring and need for evidence-based recommendations. Front Med (Lausanne). (2018) 5:302. 10.3389/fmed.2018.00302 30430111 PMC6220714

[24] Hajjar IM Grim CE George V Kotchen TA. Impact of diet on blood pressure and age-related changes in blood pressure in the US population: analysis of NHANES III. Arch Intern Med. (2001) 161:589–93. 10.1001/archinte.161.4.589 11252120

[25] Appel LJ Moore TJ Obarzanek E Vollmer WM Svetkey LP Sacks FM. A clinical trial of the effects of dietary patterns on blood pressure. DASH Collaborative Research Group. N Engl J Med. (1997) 336:1117–24. 10.1056/NEJM199704173361601 9099655

[26] Zhu K Devine A Prince RL. The effects of high potassium consumption on bone mineral density in a prospective cohort study of elderly postmenopausal women. Osteoporos Int. (2009) 20:335–40. 10.1007/s00198-008-0666-3 18575949

[27] Stevens PE Levin A. Kidney disease: improving global outcomes chronic kidney disease guideline development work group members. Evaluation and management of chronic kidney disease: synopsis of the kidney disease: improving global outcomes 2012 clinical practice guideline. Ann Intern Med. (2013). 158:825–30. 10.7326/0003-4819-158-11-201306040-00007 23732715

[28] Goraya N Munoz-Maldonado Y Simoni J Wesson DE. Fruit and vegetable treatment of chronic kidney disease-related metabolic acidosis reduces cardiovascular risk better than sodium bicarbonate. Am J Nephrol. (2019). 49:438–48. 10.1159/000500042 30995657

[29] Clinical practice guidelines for nutrition in chronic renal failure. K/DOQI, National Kidney Foundation. Am J Kidney Dis. (2000). 35(6 Suppl 2):S1–140. (Erratum in: *Am J Kidney Dis*. (2001). 38:917). 10.1053/ajkd.2000.v35.aajkd03517 10895784

[30] Fouque D Vennegoor M ter Wee P Wanner C Basci A Canaud B. Nephrol Dial Transplant. (2007) 22(Suppl 2):ii45–87. 10.1093/ndt/gfm020 17507426

[31] Kidney Disease: Improving Global Outcomes (KDIGO) CKD-MBD Update Work Group. KDIGO 2017 clinical practice guideline update for the diagnosis, evaluation, prevention, and treatment of Chronic Kidney Disease-Mineral and Bone Disorder (CKD-MBD). Kidney Int Suppl. (2011). 7:1–59. 10.1016/j.kisu.2017.04.001 (Erratum in: *Kidney Int Suppl*. (2017). 7:e1). PMC6340919 30675420

[32] Molina P Carrero JJ Bover J Chauveau P Mazzaferro S Torres PU. European Renal Nutrition (ERN) and Chronic Kidney Disease-Mineral and Bone Disorder (CKD-MBD) Working Groups of the European Renal Association-European Dialysis Transplant Association (ERA-EDTA). Vitamin D, a modulator of musculoskeletal health in chronic kidney disease. J Cachexia Sarcopenia Muscle. (2017). 8:686–701. 10.1002/jcsm.12218 28675610 PMC5659055

[33] Kelly JT Su G Zhang L Qin X Marshall S González-Ortiz A. Modifiable lifestyle factors for primary prevention of CKD: a systematic review and meta-analysis. J Am Soc Nephrol. (2021) 32:239–53. 10.1681/ASN.2020030384 32868398 PMC7894668

[34] Berg JM Tymoczko JL Stryer L. Section 23.3, The First Step in Amino Acid Degradation Is the Removal of Nitrogen. In: Biochemistry. 5th ed. New York, NY: W H Freeman. (2002). Available online at: https://www.ncbi.nlm.nih.gov/books/NBK22475/ (accessed January 15, 2021).

[35] Anatomy and Physiology. Provided by: OpenStax CNX. Available online at: http://cnx.org/contents/14fb4ad7-39a1-4eee-ab6e-3ef2482e3e22@8.25 (accessed January 15, 2021).

[36] Berg JM Tymoczko JL Stryer L. Chapter 24: The biosynthesis of amino acids biochemistry. In: Biochemistry. 5th ed. New York, NY: W H Freeman. (2002). Available online at: https://www.ncbi.nlm.nih.gov/books/NBK21178/ (accessed January 15, 2021).

[37] Berg JM Tymoczko JL Stryer L. Section 24.1, nitrogen fixation: microorganisms use ATP and a powerful reductant to reduce atmospheric nitrogen to ammonia. In: Biochemistry. 5th ed. New York, NY: W H Freeman. (2002). Available online at: https://www.ncbi.nlm.nih.gov/books/NBK22522/ (accessed January 15, 2021).

[38] Berg JM Tymoczko JL Stryer L. Section 24.2, Amino acids are made from intermediates of the citric acid cycle and other major pathways. In: Biochemistry. 5th ed. New York, NY: W H Freeman. (2002). Available online at: https://www.ncbi.nlm.nih.gov/books/NBK22459/ (accessed January 15, 2021).

[39] Palsson R Waikar SS. Renal functional reserve revisited. Adv Chronic Kidney Dis. (2018) 25:e1–8. 10.1053/j.ackd.2018.03.001 29793670

[40] Gabbai FB. Renal reserve in patients with high blood pressure. Semin Nephrol. (1995) 15:482–7. 8525152

[41] Brenner BM Cooper ME de Zeeuw D Keane WF Mitch WE Parving HH. Effects of losartan on renal and cardiovascular outcomes in patients with type 2 diabetes and nephropathy. N Engl J Med. (2001). 345:861–9. 10.1056/NEJMoa011161 11565518

[42] Helal I Fick-Brosnahan GM Reed-Gitomer B Schrier RW. Glomerular hyperfiltration: definitions, mechanisms and clinical implications. Nat Rev Nephrol. (2012) 8:293–300. 10.1038/nrneph.2012.19 22349487

[43] Kalantar-Zadeh K Kramer HM Fouque D. High-protein diet is bad for kidney health: unleashing the taboo. Nephrol Dial Transplant. (2020) 35:1–4. 10.1093/ndt/gfz216 31697325

[44] Kopple JD Coburn JW. Metabolic studies of low protein diets in uremia. Nitrogen I, and potassium. Medicine (Baltimore). (1973) 52:583–95. 10.1097/00005792-197311000-00004 4748592

[45] Tom K Young VR Chapman T Masud T Akpele L Maroni BJ. Long-term adaptive responses to dietary protein restriction in chronic renal failure. Am J Physiol. (1995) 268(4 Pt 1):E668–77. 10.1152/ajpendo.1995.268.4.E668 7733266

[46] Masud T Young VR Chapman T Maroni BJ. Adaptive responses to very low protein diets: the first comparison of ketoacids to essential amino acids. Kidney Int. (1994) 45:1182–92. 10.1038/ki.1994.157 8007590

[47] Maroni BJ Staffeld C Young VR Manatunga A Tom K. Mechanisms permitting nephrotic patients to achieve nitrogen equilibrium with a protein-restricted diet. J Clin Invest. (1997) 99:2479–87. 10.1172/JCI119432 9153292 PMC508089

[48] Kopple JD Monteon FJ Shaib JK. Effect of energy intake on nitrogen metabolism in nondialyzed patients with chronic renal failure. Kidney Int. (1986) 29:734–42. 10.1038/ki.1986.59 3702224

[49] Wang AY Kalantar-Zadeh K Fouque D Wee PT Kovesdy CP Price SR. Precision medicine for nutritional management in end-stage kidney disease and transition to dialysis. Semin Nephrol. (2018) 38:383–96. 10.1016/j.semnephrol.2018.05.008 30082058

[50] Fouque D Aparicio M. Eleven reasons to control the protein intake of patients with chronic kidney disease. Nat Clin Pract Nephrol. (2007) 3:383–92. 10.1038/ncpneph0524 17592471

[51] Robertson L Waugh N Robertson A. Protein restriction for diabetic renal disease. Cochrane Database Syst Rev. (2007) 2007:CD002181. 10.1002/14651858.CD002181.pub2 17943769 PMC8984680

[52] Fouque D Laville M Boissel JP Chifflet R Labeeuw M Zech PY. Controlled low protein diets in chronic renal insufficiency: meta-analysis. BMJ. (1992) 304:216–20. 10.1136/bmj.304.6821.216 1531426 PMC1881445

[53] Pedrini MT Levey AS Lau J Chalmers TC Wang PH. The effect of dietary protein restriction on the progression of diabetic and nondiabetic renal diseases: a meta-analysis. Ann Intern Med. (1996) 124:627–32. 10.7326/0003-4819-124-7-199604010-00002 8607590

[54] Kasiske BL Lakatua JD Ma JZ Louis TA. A meta-analysis of the effects of dietary protein restriction on the rate of decline in renal function. Am J Kidney Dis. (1998) 31:954–61. 10.1053/ajkd.1998.v31.pm9631839 9631839

[55] Fouque D Wang P Laville M Boissel JP. Low protein diets delay end-stage renal disease in non-diabetic adults with chronic renal failure. Nephrol Dial Transplant. (2000) 15:1986–92. 10.1093/ndt/15.12.1986 11096144

[56] Fouque D Laville M. Low protein diets for chronic kidney disease in non diabetic adults. Cochrane Database Syst Rev. (2009) 2009:CD001892. 10.1002/14651858.CD001892.pub3 (Update in: *Cochrane Database Syst Rev*. (2018). 10:CD001892). 19588328

[57] Rysz J Franczyk B Ciałkowska-Rysz A Gluba-Brzózka A. The effect of diet on the survival of patients with chronic kidney disease. Nutrients. (2017) 9:495. 10.3390/nu9050495 28505087 PMC5452225

[58] Hansen HP Tauber-Lassen E Jensen BR Parving HH. Effect of dietary protein restriction on prognosis in patients with diabetic nephropathy. Kidney Int. (2002) 62:220–8. 10.1046/j.1523-1755.2002.00421.x 12081581

[59] Rosman JB ter Wee PM Piers-Becht GP Sluiter WJ van der Woude FJ Meijer S. Early protein restriction in chronic renal failure. Proc Eur Dial Transplant Assoc Eur Ren Assoc. (1985) 21:567–73. 3887375

[60] Locatelli F Alberti D Graziani G Buccianti G Redaelli B Giangrande A. Prospective, randomised, multicentre trial of effect of protein restriction on progression of chronic renal insufficiency. Northern Italian Cooperative Study Group. Lancet. (1991) 337:1299–304. 10.1016/0140-6736(91)92977-a 1674294

[61] Cianciaruso B Pota A Bellizzi V Di Giuseppe D Di Micco L Minutolo R. Effect of a low- versus moderate-protein diet on progression of CKD: follow-up of a randomized controlled trial. Am J Kidney Dis. (2009) 54:1052–61. 10.1053/j.ajkd.2009.07.021 19800722

[62] Sánchez C Aranda P Planells E Galindo P Pérez de la Cruz A Larrubia M. Influence of low-protein dietetic foods consumption on quality of life and levels of B vitamins and homocysteine in patients with chronic renal failure. Nutr Hosp. (2010) 25:238–44. 10.3305/nh.2010.25.2.4274 20449532

[63] Klahr S Levey AS Beck GJ Caggiula AW Hunsicker L Kusek JW. The effects of dietary protein restriction and blood-pressure control on the progression of chronic renal disease. Modification of Diet in Renal Disease Study Group. N Engl J Med. (1994) 330:877–84. 10.1056/NEJM199403313301301 8114857

[64] Levey AS Adler S Caggiula AW England BK Greene T Hunsicker LG. Effects of dietary protein restriction on the progression of advanced renal disease in the Modification of Diet in Renal Disease Study. Am J Kidney Dis. (1996) 27:652–63. 10.1016/s0272-6386(96)90099-2 8629624

[65] Coggins CH Dwyer JT Greene T Petot G Snetselaar LG Van Lente F. Serum lipid changes associated with modified protein diets: results from the feasibility phase of the Modification of Diet in Renal Disease Study. Am J Kidney Dis. (1994) 23:514–23. 10.1016/s0272-6386(12)80372-6 8154486

[66] Bellizzi V Di Iorio BR De Nicola L Minutolo R Zamboli P Trucillo P. Very low protein diet supplemented with ketoanalogs improves blood pressure control in chronic kidney disease. Kidney Int. (2007). 71:245–51. 10.1038/sj.ki.5001955 17035939

[67] Feiten SF Draibe SA Watanabe R Duenhas MR Baxmann AC Nerbass FB. Short-term effects of a very-low-protein diet supplemented with ketoacids in nondialyzed chronic kidney disease patients. Eur J Clin Nutr. (2005) 59:129–36. 10.1038/sj.ejcn.1602050 15354199

[68] Herselman MG Albertse EC Lombard CJ Swanepoel CR Hough FS. Supplemented low-protein diets–are they superior in chronic renal failure? S Afr Med J. (1995) 85:361–5. 7638685

[69] Jiang N Qian J Sun W Lin A Cao L Wang Q. Better preservation of residual renal function in peritoneal dialysis patients treated with a low-protein diet supplemented with keto acids: a prospective, randomized trial. Nephrol Dial Transplant. (2009) 24:2551–8. 10.1093/ndt/gfp085 19258386

[70] Jungers P Chauveau P Ployard F Lebkiri B Ciancioni C Man NK. Comparison of ketoacids and low protein diet on advanced chronic renal failure progression. Kidney Int Suppl. (1987) 22:S67–71. 3323621

[71] Kopple JD Levey AS Greene T Chumlea WC Gassman JJ Hollinger DL. Effect of dietary protein restriction on nutritional status in the Modification of Diet in Renal Disease Study. Kidney Int. (1997) 52:778–91. 10.1038/ki.1997.395 9291200

[72] Li H Long Q Shao C Fan H Yuan L Huang B. Effect of short-term low-protein diet supplemented with keto acids on hyperphosphatemia in maintenance hemodialysis patients. Blood Purif. (2011) 31:33–40. 10.1159/000321376 21135547

[73] Malvy D Maingourd C Pengloan J Bagros P Nivet H. Effects of severe protein restriction with ketoanalogues in advanced renal failure. J Am Coll Nutr. (1999) 18:481–6. 10.1080/07315724.1999.10718887 10511331

[74] Menon V Wang X Greene T Beck GJ Kusek JW Selhub J. Homocysteine in chronic kidney disease: effect of low protein diet and repletion with B vitamins. Kidney Int. (2005) 67:1539–46. 10.1111/j.1523-1755.2005.00234.x 15780109

[75] Mircescu G Gârneată L Stancu SH Căpuşă C. Effects of a supplemented hypoproteic diet in chronic kidney disease. J Ren Nutr. (2007) 17:179–88. 10.1053/j.jrn.2006.12.012 17462550

[76] Prakash S Pande DP Sharma S Sharma D Bal CS Kulkarni H. Randomized, double-blind, placebo-controlled trial to evaluate efficacy of ketodiet in predialytic chronic renal failure. J Ren Nutr. (2004) 14:89–96. 10.1053/j.jrn.2004.01.008 15060873

[77] Brenner BM Lawler EV Mackenzie HS. The hyperfiltration theory: a paradigm shift in nephrology. Kidney Int. (1996) 49:1774–7. 10.1038/ki.1996.265 8743495

[78] Rosenberg ME Salahudeen AK Hostetter TH. Dietary protein and the renin-angiotensin system in chronic renal allograft rejection. Kidney Int Suppl. (1995) 52:S102–6. 8587269

[79] Kalantar-Zadeh K Moore LW Tortorici AR Chou JA St-Jules DE Aoun A. North American experience with low protein diet for non-dialysis-dependent chronic kidney disease. BMC Nephrol. (2016) 17:90. 10.1186/s12882-016-0304-9 27435088 PMC4952055

[80] Bellizzi V Cupisti A Locatelli F Bolasco P Brunori G Cancarini G. Low-protein diets for chronic kidney disease patients: the Italian experience. BMC Nephrol. (2016) 17:77. 10.1186/s12882-016-0280-0 27401096 PMC4939662

[81] Apetrii M Timofte D Voroneanu L Covic A. Nutrition in chronic kidney disease-the role of proteins and specific diets. Nutrients. (2021) 13:956. 10.3390/nu13030956 33809492 PMC7999704

[82] Milas NC Nowalk MP Akpele L Castaldo L Coyne T Doroshenko L. Factors associated with adherence to the dietary protein intervention in the Modification of Diet in Renal Disease Study. J Am Diet Assoc. (1995) 95:1295–300. 10.1016/s0002-8223(95)00340-1 7594126

[83] Paes-Barreto JG Silva MI Qureshi AR Bregman R Cervante VF Carrero JJ. Can renal nutrition education improve adherence to a low-protein diet in patients with stages 3 to 5 chronic kidney disease? J Ren Nutr. (2013) 23:164–71. 10.1053/j.jrn.2012.10.004 23194841

[84] Dolecek TA Olson MB Caggiula AW Dwyer JT Milas NC Gillis BP. Registered dietitian time requirements in the Modification of Diet in Renal Disease Study. J Am Diet Assoc. (1995) 95:1307–12. 10.1016/s0002-8223(95)00342-8 7594128

[85] Pisani A Riccio E Bellizzi V Caputo DL Mozzillo G Amato M. 6-tips diet: a simplified dietary approach in patients with chronic renal disease. A clinical randomized trial. Clin Exp Nephrol. (2016). 20:433–42. 10.1007/s10157-015-1172-5 26453483

[86] D'Alessandro C Piccoli GB Calella P Brunori G Pasticci F Egidi MF. “Dietaly”: practical issues for the nutritional management of CKD patients in Italy. BMC Nephrol. (2016). 17:102. 10.1186/s12882-016-0296-5 27473183 PMC4966713

[87] Bellizzi V Di Iorio BR Brunori G De Nicola L Minutolo R Conte G. Assessment of nutritional practice in Italian chronic kidney disease clinics: a questionnaire-based survey. J Ren Nutr. (2010) 20:82–90. 10.1053/j.jrn.2009.05.001 19616451

[88] Knight EL Stampfer MJ Hankinson SE Spiegelman D Curhan GC. The impact of protein intake on renal function decline in women with normal renal function or mild renal insufficiency. Ann Intern Med. (2003) 138:460–7. 10.7326/0003-4819-138-6-200303180-00009 12639078

[89] Garneata L Stancu A Dragomir D Stefan G Mircescu G. Ketoanalogue-supplemented vegetarian very low-protein diet and CKD progression. J Am Soc Nephrol. (2016) 27:2164–76. 10.1681/ASN.2015040369 26823552 PMC4926970

[90] Scialla JJ Appel LJ Astor BC Miller ER III Beddhu S Woodward M Parekh RS Anderson CA. African American Study of Kidney Disease and Hypertension Study Group. Net endogenous acid production is associated with a faster decline in GFR in African Americans. Kidney Int. (2012). 82:106–12. 10.1038/ki.2012.82 22475819 PMC3540413

[91] Raphael KL Wei G Baird BC Greene T Beddhu S. Higher serum bicarbonate levels within the normal range are associated with better survival and renal outcomes in African Americans. Kidney Int. (2011) 79:356–62. 10.1038/ki.2010.388 20962743 PMC5241271

[92] Lew QJ Jafar TH Koh HW Jin A Chow KY Yuan JM. Red meat intake and risk of ESRD. J Am Soc Nephrol. (2017) 28:304–12. 10.1681/ASN.2016030248 27416946 PMC5198288

[93] Toeller M Buyken A Heitkamp G Brämswig S Mann J Milne R. Protein intake and urinary albumin excretion rates in the EURODIAB IDDM Complications Study. Diabetologia. (1997) 40:1219–26. 10.1007/s001250050810 9349605

[94] Nettleton JA Steffen LM Palmas W Burke GL Jacobs DR Jr. Associations between microalbuminuria and animal foods, plant foods, and dietary patterns in the Multiethnic Study of Atherosclerosis. Am J Clin Nutr. (2008). 87:1825–36. 10.1093/ajcn/87.6.1825 18541574 PMC2503276

[95] Patel KP Luo FJ Plummer NS Hostetter TH Meyer TW. The production of p-cresol sulfate and indoxyl sulfate in vegetarians versus omnivores. Clin J Am Soc Nephrol. (2012) 7:982–8. 10.2215/CJN.12491211 22490877 PMC3362314

[96] Wu IW Hsu KH Lee CC Sun CY Hsu HJ Tsai CJ. p-Cresyl sulphate and indoxyl sulphate predict progression of chronic kidney disease. Nephrol Dial Transplant. (2011) 26:938–47. 10.1093/ndt/gfq580 20884620 PMC3042976

[97] Motojima M Hosokawa A Yamato H Muraki T Yoshioka T. Uremic toxins of organic anions up-regulate PAI-1 expression by induction of NF-kappaB and free radical in proximal tubular cells. Kidney Int. (2003) 63:1671–80. 10.1046/j.1523-1755.2003.00906.x 12675842

[98] Niwa T Ise M. Indoxyl sulfate, a circulating uremic toxin, stimulates the progression of glomerular sclerosis. J Lab Clin Med. (1994) 124:96–104. 8035108

[99] Sun CY Chang SC Wu MS. Uremic toxins induce kidney fibrosis by activating intrarenal renin-angiotensin-aldosterone system associated epithelial-to-mesenchymal transition. PLoS ONE. (2012) 7:e34026. 10.1371/journal.pone.0034026 22479508 PMC3316590

[100] Moe SM Zidehsarai MP Chambers MA Jackman LA Radcliffe JS Trevino LL. Vegetarian compared with meat dietary protein source and phosphorus homeostasis in chronic kidney disease. Clin J Am Soc Nephrol. (2011) 6:257–64. 10.2215/CJN.05040610 21183586 PMC3052214

[101] Scialla JJ Appel LJ Astor BC Miller ER 3rd Beddhu S Woodward M. Estimated net endogenous acid production and serum bicarbonate in African Americans with chronic kidney disease. Clin J Am Soc Nephrol. (2011) 6:1526–32. 10.2215/CJN.00150111 21700817 PMC3552445

[102] Chen X Wei G Jalili T Metos J Giri A Cho ME. The associations of plant protein intake with all-cause mortality in CKD. Am J Kidney Dis. (2016) 67:423–30. 10.1053/j.ajkd.2015.10.018 26687923 PMC4769135

[103] Kelly JT Carrero JJ. Dietary sources of protein and chronic kidney disease progression: the proof may be in the pattern. J Ren Nutr. (2017) 27:221–4. 10.1053/j.jrn.2017.04.001 28549571

[104] Carrero JJ González-Ortiz A Avesani CM Bakker SJL Bellizzi V Chauveau P. Plant-based diets to manage the risks and complications of chronic kidney disease. Nat Rev Nephrol. (2020) 16:525–42. 10.1038/s41581-020-0297-2 32528189

[105] Suckling RJ He FJ Macgregor GA. Altered dietary salt intake for preventing and treating diabetic kidney disease. Cochrane Database Syst Rev. (2010) 2010:CD006763. 10.1002/14651858.CD006763.pub2 21154374

[106] Graudal NA Hubeck-Graudal T Jürgens G. Effects of low-sodium diet vs. high-sodium diet on blood pressure, renin, aldosterone, catecholamines, cholesterol, and triglyceride (Cochrane Review). Am J Hypertens. (2012) 25:1–15. 10.1038/ajh.2011.210 22068710

[107] Essig M Escoubet B de Zuttere D Blanchet F Arnoult F Dupuis E. Cardiovascular remodelling and extracellular fluid excess in early stages of chronic kidney disease. Nephrol Dial Transplant. (2008) 23:239–48. 10.1093/ndt/gfm542 17704109

[108] Schweda F. Salt feedback on the renin-angiotensin-aldosterone system. Pflugers Arch. (2015) 467:565–76. 10.1007/s00424-014-1668-y 25502115

[109] Schmieder RE Mann JF Schumacher H Gao P Mancia G Weber MA. ONTARGET Investigators. Changes in albuminuria predict mortality and morbidity in patients with vascular disease. J Am Soc Nephrol. (2011). 22:1353–64. 10.1681/ASN.2010091001 21719791 PMC3137583

[110] McMahon EJ Bauer JD Hawley CM Isbel NM Stowasser M Johnson DW. A randomized trial of dietary sodium restriction in CKD. J Am Soc Nephrol. (2013) 24:2096–103. 10.1681/ASN.2013030285 24204003 PMC3839553

[111] van den Berg E Geleijnse JM Brink EJ van Baak MA Homan van der Heide JJ Gans RO. Sodium intake and blood pressure in renal transplant recipients. Nephrol Dial Transplant. (2012) 27:3352–9. 10.1093/ndt/gfs069 22499024

[112] Slagman MC Waanders F Hemmelder MH Woittiez AJ Janssen WM Lambers Heerspink HJ. Moderate dietary sodium restriction added to angiotensin converting enzyme inhibition compared with dual blockade in lowering proteinuria and blood pressure: randomised controlled trial. BMJ. (2011). 343:d4366. 10.1136/bmj.d4366 21791491 PMC3143706

[113] Konishi Y Okada N Okamura M Morikawa T Okumura M Yoshioka K. Sodium sensitivity of blood pressure appearing before hypertension and related to histological damage in immunoglobulin a nephropathy. Hypertension. (2001) 38:81–5. 10.1161/01.hyp.38.1.81 11463764

[114] De Brito-Ashurst Perry L Sanders TA Thomas JE Dobbie H Yaqoob MM. PP077-MON a dietitian's role in the management of blood pressure: results of a randomised controlled trial in british bangladeshi chronic kidney disease patients. Clin Nutr Suppl. (2012) 7:168–9. 10.1016/S1744-1161(12)70416-7

[115] Vogt L Waanders F Boomsma F de Zeeuw D Navis G. Effects of dietary sodium and hydrochlorothiazide on the antiproteinuric efficacy of losartan. J Am Soc Nephrol. (2008) 19:999–1007. 10.1681/ASN.2007060693 18272844 PMC2386733

[116] St-Jules DE Rozga MR Handu D Carrero JJ. Effect of phosphate-specific diet therapy on phosphate levels in adults undergoing maintenance hemodialysis: a systematic review and meta-analysis. Clin J Am Soc Nephrol. (2020) 16:107–20. 10.2215/CJN.09360620 33380474 PMC7792658

[117] Palmer SC Hayen A Macaskill P Pellegrini F Craig JC Elder GJ. Serum levels of phosphorus, parathyroid hormone, and calcium and risks of death and cardiovascular disease in individuals with chronic kidney disease: a systematic review and meta-analysis. JAMA. (2011) 305:1119–27. 10.1001/jama.2011.308 21406649

[118] Górriz JL Molina P Cerverón MJ Vila R Bover J Nieto J. Vascular calcification in patients with nondialysis CKD over 3 years. Clin J Am Soc Nephrol. (2015) 10:654–66. 10.2215/CJN.07450714 25770175 PMC4386255

[119] Molina P Molina MD Pallardó LM Torralba J Escudero V Álvarez L. Disorders in bone-mineral parameters and the risk of death in persons with chronic kidney disease stages 4 and 5: the PECERA study. J Nephrol. (2021). 10.1007/s40620-020-00916-9. [Epub ahead of print]. 33394344

[120] Bellasi A Mandreoli M Baldrati L Corradini M Di Nicolò P Malmusi G. Chronic kidney disease progression and outcome according to serum phosphorus in mild-to-moderate kidney dysfunction. Clin J Am Soc Nephrol. (2011) 6:883–91. 10.2215/CJN.07810910 21393493 PMC3069383

[121] Zoccali C Ruggenenti P Perna A Leonardis D Tripepi R Tripepi G. Phosphate may promote CKD progression and attenuate renoprotective effect of ACE inhibition. J Am Soc Nephrol. (2011). 22:1923–30. 10.1681/ASN.2011020175 21852581 PMC3279951

[122] Chartsrisak K Vipattawat K Assanatham M Nongnuch A Ingsathit A Domrongkitchaiporn S. Mineral metabolism and outcomes in chronic kidney disease stage 2–4 patients. BMC Nephrol. (2013) 14:14. 10.1186/1471-2369-14-14 23324569 PMC3551685

[123] Caravaca F Villa J García de Vinuesa E Martínez del Viejo C Martínez Gallardo R Macías R. Relationship between serum phosphorus and the progression of advanced chronic kidney disease. Nefrologia. (2011) 31:707–15. In English, Spanish. 10.3265/Nefrologia.pre2011.Sep.11089 22130287

[124] Bover J Molina P Ureña-Torres P Arenas MD. Feasible low-phosphorus dietary patterns in maintenance hemodialysis patients: need for original research. Kidney Int Rep. (2020) 5:1845–7. 10.1016/j.ekir.2020.09.008 33165412 PMC7610001

[125] Merhi B Shireman T Carpenter MA Kusek JW Jacques P Pfeffer M. Serum phosphorus and risk of cardiovascular disease, all-cause mortality, or Graft failure in kidney transplant recipients: an ancillary study of the FAVORIT Trial Cohort. Am J Kidney Dis. (2017) 70:377–85. 10.1053/j.ajkd.2017.04.014 28579423 PMC5704919

[126] Stevens KK Morgan IR Patel RK Geddes CC Mark PB Jardine AG. Serum phosphate and outcome at one year after deceased donor renal transplantation. Clin Transplant. (2011) 25:E199–204. 10.1111/j.1399-0012.2011.01400.x 21303413

[127] Jeon HJ Kim YC Park S Kim CT Ha J Han DJ. Association of serum phosphorus concentration with mortality and graft failure among kidney transplant recipients. Clin J Am Soc Nephrol. (2017) 12:653–62. 10.2215/CJN.07090716 28159828 PMC5383385

[128] Vivante A Golan E Tzur D Leiba A Tirosh A Skorecki K. Body mass index in 1.2 million adolescents and risk for end-stage renal disease. Arch Intern Med. (2012) 172:1644–50. 10.1001/2013.jamainternmed.85 23108588 PMC4941233

[129] Eknoyan G. Obesity and chronic kidney disease. Nefrologia. (2011). 31:397–403. 10.3265/Nefrologia.pre2011.May.10963 21623393

[130] Docherty NG le Roux CW. Bariatric surgery for the treatment of chronic kidney disease in obesity and type 2 diabetes mellitus. Nat Rev Nephrol. (2020) 16:709–20. 10.1038/s41581-020-0323-4 32778788

[131] Chang AR Grams ME Navaneethan SD. Bariatric surgery and kidney-related outcomes. Kidney Int Rep. (2017) 2:261–70. 10.1016/j.ekir.2017.01.010 28439568 PMC5399773

[132] Ikizler TA Robinson-Cohen C Ellis C Headley SAE Tuttle K Wood RJ. Metabolic effects of diet and exercise in patients with moderate to severe CKD: a randomized clinical trial. J Am Soc Nephrol. (2018) 29:250–9. 10.1681/ASN.2017010020 29038285 PMC5748901

[133] Chagnac A Weinstein T Herman M Hirsh J Gafter U Ori Y. The effects of weight loss on renal function in patients with severe obesity. J Am Soc Nephrol. (2003) 14:1480–6. 10.1097/01.asn.0000068462.38661.89 12761248

[134] MacLaughlin HL Sarafidis PA Greenwood SA Campbell KL Hall WL Macdougall IC. Compliance with a structured weight loss program is associated with reduced systolic blood pressure in obese patients with chronic kidney disease. Am J Hypertens. (2012) 25:1024–9. 10.1038/ajh.2012.80 22717545

[135] Praga M Hernández E Andrés A León M Ruilope LM Rodicio JL. Effects of body-weight loss and captopril treatment on proteinuria associated with obesity. Nephron. (1995) 70:35–41. 10.1159/000188541 7617115

[136] Morales E Valero MA León M Hernández E Praga M. Beneficial effects of weight loss in overweight patients with chronic proteinuric nephropathies. Am J Kidney Dis. (2003) 41:319–27. 10.1053/ajkd.2003.50039 12552492

[137] Salmean YA Segal MS Palii SP Dahl WJ. Fiber supplementation lowers plasma p-cresol in chronic kidney disease patients. J Ren Nutr. (2015) 25:316–20. 10.1053/j.jrn.2014.09.002 25446837 PMC4646076

[138] Salmean YA Segal MS Langkamp-Henken B Canales MT Zello GA Dahl WJ. Foods with added fiber lower serum creatinine levels in patients with chronic kidney disease. J Ren Nutr. (2013) 23:e29–32. 10.1053/j.jrn.2012.04.002 22739658

[139] Guida B Germanò R Trio R Russo D Memoli B Grumetto L. Effect of short-term synbiotic treatment on plasma p-cresol levels in patients with chronic renal failure: a randomized clinical trial. Nutr Metab Cardiovasc Dis. (2014) 24:1043–9. 10.1016/j.numecd.2014.04.007 24929795

[140] Huang X Jiménez-Moleón JJ Lindholm B Cederholm T Arnlöv J Risérus U. Mediterranean diet, kidney function, and mortality in men with CKD. Clin J Am Soc Nephrol. (2013) 8:1548–55. 10.2215/CJN.01780213 23744002 PMC3805069

[141] Osté MCJ Gomes-Neto AW Corpeleijn E Gans ROB de Borst MH van den Berg E. Dietary Approach to Stop Hypertension (DASH) diet and risk of renal function decline and all-cause mortality in renal transplant recipients. Am J Transplant. (2018) 18:2523–33. 10.1111/ajt.14707 29464830 PMC6175360

[142] Lin J Fung TT Hu FB Curhan GC. Association of dietary patterns with albuminuria and kidney function decline in older white women: a subgroup analysis from the Nurses' Health Study. Am J Kidney Dis. (2011) 57:245–54. 10.1053/j.ajkd.2010.09.027 21251540 PMC3026604

[143] Kelly JT Palmer SC Wai SN Ruospo M Carrero JJ Campbell KL. Healthy dietary patterns and risk of mortality and ESRD in CKD: a meta-analysis of cohort studies. Clin J Am Soc Nephrol. (2017) 12:272–9. 10.2215/CJN.06190616 27932391 PMC5293335

[144] Mekki K Bouzidi-bekada N Kaddous A Bouchenak M. Mediterranean diet improves dyslipidemia and biomarkers in chronic renal failure patients. Food Funct. (2010) 1:110–5. 10.1039/c0fo00032a 21776461

[145] Goraya N Simoni J Jo CH Wesson DE. Treatment of metabolic acidosis in patients with stage 3 chronic kidney disease with fruits and vegetables or oral bicarbonate reduces urine angiotensinogen and preserves glomerular filtration rate. Kidney Int. (2014) 86:1031–8. 10.1038/ki.2014.83 24694986

[146] Chauveau P Aparicio M Bellizzi V Campbell K Hong X Johansson L. Mediterranean diet as the diet of choice for patients with chronic kidney disease. Nephrol Dial Transplant. (2018). 33:725–35. 10.1093/ndt/gfx085 29106612

